# Towards Systems Biology of Mycotoxin Regulation

**DOI:** 10.3390/toxins5040675

**Published:** 2013-04-18

**Authors:** Rajagopal Subramaniam, Christof Rampitsch

**Affiliations:** 1 Eastern Cereal & Oilseed Research Centre, Agriculture & Agri-Food Canada, Ottawa, ON K1A 0C6, Canada; 2 Cereal Research Centre, Agriculture & Agri-Food Canada, Winnipeg, MB R3T 2M9, Canada

**Keywords:** synthetic genetic array, *Saccharomyces cerevisiae*, mycotoxin, chemical genetics

## Abstract

Systems biology is a scientific approach that integrates many scientific disciplines to develop a comprehensive understanding of biological phenomena, thus allowing the prediction and accurate simulation of complex biological behaviors. It may be presumptuous to write about toxin regulation at the level of systems biology, but the last decade of research is leading us closer than ever to this approach. Past research has delineated multiple levels of regulation in the pathways leading to the biosynthesis of secondary metabolites, including mycotoxins. At the top of this hierarchy, the global or master transcriptional regulators perceive various environmental cues such as climatic conditions, the availability of nutrients, and the developmental stages of the organism. Information accumulated from various inputs is integrated through a complex web of signalling networks to generate the eventual outcome. This review will focus on adapting techniques such as chemical and other genetic tools available in the model system *Saccharomyces cerevisiae*, to disentangle the various biological networks involved in the biosynthesis of mycotoxins in the *Fusarium* spp.

## 1. Biological Networks in the Context of Mycotoxin Biosynthesis

Through analyses of many fungal genomes confirmed by genetic experiments, we now understand that the genes required for the biosynthesis of the majority of secondary metabolites occur in clusters [1,2,3,4]. Additionally, the activity of regulatory genes within these clusters is closely associated with biological networks involved in many aspects of normal cellular function [1,2]. For example, in *Fusarium* spp. the biosynthesis of mycotoxins such as deoxynivalenol (DON) and T2 toxin is linked to the signal transduction network associated with oxidative stress and to cellular networks responsive to pH, nitrogen and carbon sources, and to other environmental signals such as light [5,6,7,8,9,10]. Although genetic experiments confirm the role of these diverse networks in mycotoxin production, they do not parse the components specific to the mycotoxin pathway. Since changes in pH or the addition of H_2_O_2_ will have broad repercussions on cellular functions not limited to mycotoxin production, it is imperative that we initially identify components in each of these networks that specifically regulate the mycotoxin pathway(s).

## 2. *Saccharomyces cerevisiae* as a Model System to Identify Components in Networks in *Fusarium*

Our current understanding of cellular networks has emerged from studies of the budding yeast *S. cerevisiae* [11]. The collection of deletion strains for all the known genes in yeast enables one to screen for mutants defective against a variety of environmental insults, including growth conditions and fungicide treatments [12]. Given that ~85% of all yeast genes can be functionally compensated by other genes, action of a second site mutation that either suppresses or enhances the original mutant phenotype has greatly advanced our knowledge of relationships between genes and pathways [11]. This methodology implemented on a larger scale, commonly referred to as synthetic genetic array (SGA) analysis has enabled the mapping of synthetic lethal genetic interactions in yeast [12]. The resulting genetic interaction profiles revealed a functional map of the cell in which genes of similar biological processes are clustered together. Importantly, genes that could not be annotated previously through single gene mutations are now able to be associated with a specific biological process. 

To complement the synthetic lethal screen where a combination of two mutants leads to cell death, SGA analysis can also be adapted to explore gain-of-function phenotypes. In this scenario, the overexpression of a protein may not affect fitness in a wild-type cell, but in a mutant strain, which lacks the interacting protein, fitness may be compromised [11].This technique termed synthetic dosage lethal (SDL) screens is being used to dissect biological process such as enzyme-substrate relationships identified by the synthetic lethal screens. More than 5000 genes were overexpressed in the yeast background with the mutation in the cyclin-dependent kinase gene, *PHO85* [13]. Analysis revealed more than 60 synthetic dosage interactions and identified four new substrates. Additionally, the screen linked two distinct cellular signalling mechanisms, like calcium and cell cycle signalling to this kinase pathway [13]. Deployment of SDL screens have proved fruitful to dissect specific biological processes related to DNA replication, chromosome segregation and proteolytic pathways [14,15,16]. 

**Figure 1:**
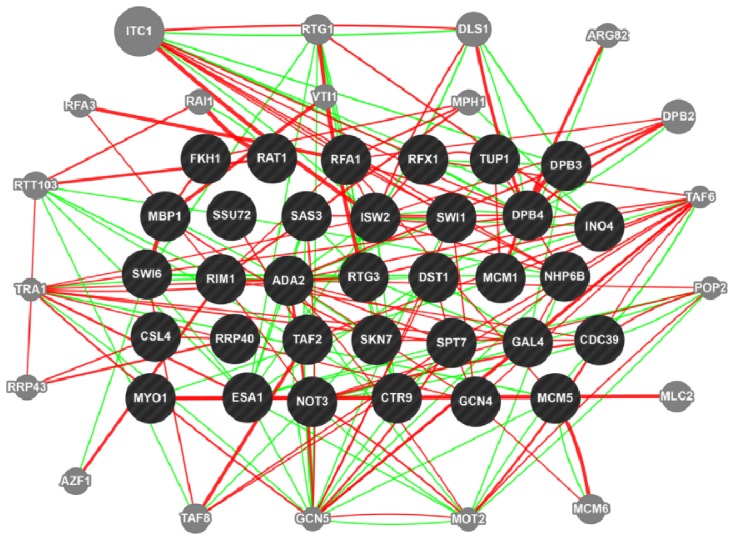
**Output of GeneMANIA**: Display of genetic (green lines) and physical (red lines) relationships between yeast orthologues of *F. graminearum* transcription factors (dark shaded circles) and other yeast genes (grey shaded circles).

Comparative analysis between *S. cerevisiae* and *F. graminearum* genomes revealed that ~4,000 genes in yeast have homologues in *F. graminearum* (e-values ≤ 1E-5) [17]. This represents ~66% of the yeast genome and incorporates biological and signalling networks associated with environmental stresses and normal cellular functions. Such significant conservation between these two organisms also allows us to use the large number of genetic and computational tools developed in the yeast system. This is best exemplified by the study involving transcription factors in *F. graminearum* in which transcription factors in *F.graminearum* were categorized by various phenotypes through large-scale deletion analyses, including those unable to produce the mycotoxin DON. With the use of a yeast protein-protein interaction database, the authors were able to construct a protein interaction map of *Fusarium* transcription factors [18]. To demonstrate the power of yeast genetic tools, we entered the yeast orthologues of *Fusarium* transcription factors involved in DON production into GeneMANIA, a functional association data analysis tool that links proteins to biological pathways (http://www.genemania.org). The output of this analysis as shown in Figure 1 demonstrates the utility and power of this tool. The dark shaded circles represent yeast orthologues of *Fusarium* transcription factors identified by Son *et al.* [18]. 

The light shaded circles in the periphery represent proteins with new genetic (green lines) and direct interactions (red) with the transcription factors. These new interacting proteins are associated with distinct cellular functions. Examples include GCN5 and TRA1 which are histone acetyl transferases involved in RNA polymerase II-dependent transcriptional regulation of ~10% of yeast genes VTI, which is associated with the SNARE complex involved in vacuolar sorting and ITC1, which is part of a chromatin remodelling complex required for repression of early meiotic genes [19,20,21]. Mutational analysis of these new interacting partners in *F. graminearum* can confirm the link between these genes and DON biosynthesis. Thus, a genetic screen in *F. graminearum* linking transcription factors to DON biosynthesis can be integrated into the overall cellular network.

Recently, a large scale mutational analysis of kinases in *F. graminearum* revealed links between several kinases and DON biosynthesis [22].Since orthologues of some of the kinases are present in yeast, a synthetic dosage lethal screen could identify substrates of these kinases. These could be introgressed into the network (Figure 1) to generate new biological networks associated with the DON pathways.

## 3. Chemogenomics to Decipher Mycotoxin Pathway in *Fusarium*

Complementary to SGA analysis, the use of small molecule inhibitors has recently emerged as a useful tool to study biological processes. Advantages of a chemical over a genetic screen are apparent. First, advances in the synthesis and commercial availability of combinatorial libraries of diverse skeletal structures along with high throughput screening has led to the identification of many molecules targeting specific pathways or networks [23,24,25]. Second, small molecules could interact with proteins or components of a signalling pathway in a reversible manner; this is especially important if essential genes are studied. Moreover, orthologous and paralogous protein functions could be differentially targeted, thus enabling a comparison between species. Lastly, precise temporal control can be exercised. For example, it is known that various developmental stages related to nutrition or environmental cues affect secondary metabolite production in many fungi [26,27]. Large scale mutant screens in *F. graminearum* have identified many genes overlapping many of these functions, underscoring the redundancies of genetic pathways (up to 85% of the genes are considered to be non-essential for yeast viability) [18,22]. The use of small molecules in a chemical screen can target specific components in the nutrition and developmental networks and thus disentangle the various functional requirements for secondary metabolite production. 

In one of the early applications of this technique, twelve inhibitory compounds including fungicides such as benomyl and fluconazole were used in a yeast chemical-genetics screen [28]. The screen identified 62 deletion strains that were sensitive to at least four of the compounds. Several of these mutants were subjected to a synthetic lethal screen. The overlap between the chemical screen and the synthetic lethal screen datasets not only provided corroborative evidence, but also implicated uncharacterized genes in specific roles [28]. A similar strategy could be employed to delineate pathways responsible for DON biosynthesis in *F. graminearum*. Since this fungus is amenable to growth in liquid culture and adaptable to high-throughput screens, a chemical library could be used at various developmental stages and different nutritional and environmental regimens, suited to induce DON production [29]. Once the compounds that mitigate or promote DON production have been identified, yeast homozygous deletion arrays can be used to identify signaling pathways. Moreover, heterozygous yeast deletion mutants grown competitively with the chemical compounds can be used to identify specific targets [12]. Thus, the use of chemicals to probe genetic interactions, combined with yeast SGA analysis can enable us to decipher specific pathways or networks used for DON synthesis. 

## 4. Proteomics to Identify Components of a Cellular Network

The aforementioned genetic tools in yeast such as SGA analysis have proven very useful to ascertain genetic interactions in organisms that are intractable or where resources are not yet available. A chemical genetics screen, on the other hand, can be used directly in many cell-based systems, including *F. graminearum* to obtain discrete functional information on those chemicals that modulate pathways responsible for DON biosynthesis. Moreover, the screen complemented with the yeast SGA analysis can yield valuable information with regards to targets of the chemical compounds [12]. Studies show that targets from chemogenetic screens can be categorized into discrete modules or local networks with specific cellular functions, such as metabolism and transcription regulation [30,31]. Evidence also suggests that components in these local networks also interact physically [11]. It follows then that studies with protein-protein interaction (PPI) data will improve our understanding of both functional modules and how they connect with each other in a global cellular network. 

Although large scale experimental PPI studies, similar to the yeast two-hybrid system have not been done with *Fusarium* proteins, a PPI database for *F. graminearum* has been curated based on an interaction-ortholog (interolog) approach [32,33]. The INPARANOID software was used to identify *Fusarium* protein orthologues from seven species and was used to predict more than 200,000 interactions with ~7,000 *Fusarium* proteins (http://csb.shu.edu.cn/fppi) [33,34]. As mentioned previously, physical interaction between proteins is observed only if they share biochemical functions. Therefore, it is unreasonable to expect functional modules responsible for DON production in this database, because such modules have evolved uniquely in *Fusarium* spp. This was confirmed by a query in this database with Tri6 and Tri10, the two regulatory proteins central to DON biosynthesis in *F. graminearum*, which did not identify any interacting proteins. Hence, it is imperative that proteins involved in DON synthesis are identified and functional modules unique to DON biosynthesis are created. In this section, we will outline some of the methods that are being used to identify proteins that are likely involved in the DON biosynthesis pathway. 

In *F. graminearum*, a host-free system has been established to induce DON, therefore allowing various proteomics tools to be used to identify proteins associated with DON production [29]. All of the enzymes involved in DON biosynthesis have been identified, along with some of the proteins which regulate the production of this mycotoxin; however, the regulatory events, including post-translational modifications (PTMs), remain largely unexplored. Since the language of cell-signalling is embedded in PTMs, a proteomic approach with mass spectrometry-based identification of modified peptides is the method of choice [35]. Strategies for high-throughput phosphoproteome analyses are well documented [36]. Amoutzias and colleagues recently summarized and analyzed 12 publicly available *S. cerevisiae* datasets comprising over 2,000 phosphoproteins and almost 10,000 phosphorylation sites [37]. Protein-protein interactions of the yeast phosphoproteome are now also beginning to be described [38,39]. A high-throughput phosphoproteome *F. graminearum* during DON induction has recently been described, but this has not been incorporated into the larger PPI dataset [33,40]. 

Other PTMs, such as redox modifications of proteins are less studied, but tools to study them are becoming widely available. Proteins with exposed cysteine residues, which monitor the cell’s redox status through reversible oxidation/reduction, have been shown to affect cellular processes such as sexual structure development in *Aspergillus* spp. [41]. In *F. graminearum*, exogenous application of H_2_O_2_ induces DON production, suggesting that redox regulation is likely involved in this process [5]. One approacht has been to analyze the redox proteome through the covalent labelling of cysteine with the fluorescent label monobromobimane, which attaches to free cysteines on proteins [42]. After the separation of proteins by two-dimensional gel electrophoresis, fluorescence ratios can be calculated to determine the incorporation of the label under different experimental conditions, *i.e.*, between oxidized and reduced states of the protein. This is followed by mass spectrometry to identify peptides with the modified cysteine. Through the use of these PTM tools and mutational studies, a comprehensive database can be compiled of proteins involved in DON biosynthesis. 

## 5. Concluding Remarks

It is evident that the early foundations for systems biology studies in *F. graminearum* are in place. Before the promise of systems biology studies of mycotoxin regulation can be fully realized, however, strategies to integrate the multiple available databases have to be developed. Nonetheless, we can take comfort in the fact that many of these challenges have already been overcome in other model organisms and it is now up to the *Fusarium* community to embrace the systems approach.
